# The Relation of Pulmonary Hemorrhage to Phthisis

**Published:** 1870-04

**Authors:** 


### The Relation of Pulmonary Hemorrhage to Phthisis.

Dr. Felix Niemeyer has a paper upon this subject in Nos. 18 and 19 of the *Berliner Klinische Wochenschrift*, 1869. The following are his conclusions:

1. Not all persons, by any means, who suffer from capillary, bronchial, or pulmonary hemorrhage, are or become consumptive.

2. Consumption not rarely follows up capillary, bronchial, and pulmonary hemorrhages, but there is no generic connection between the hemorrhages and the pneumonic processes which generally form the starting point of consumption. The persons who are predisposed to those hemorrhages, have also a predisposition to the above named inflammatory processes.

3. Capillary, bronchial, and pulmonary hemorrhages not infrequently lay the foundation of consumption in persons in whose lungs neither tubercles nor pneumonic centres are present; and when they do so, it is through cheesy metamorphosis of the blood remaining in the alveoli of the lungs, and the product of the inflammation caused by its pressure.

4. In the same way bronchial and pulmonary hemorrhages hasten the course of an already existing phthisis.

6. In rare isolated cases the hæmoptysis is not the cause, but the result of pneumonia process, which finally lead to consumption. Such cases are readily recognized, inasmuch as the hemorrhage is usually accompanied with, or preceded by, fever and other inflammatory phenomena.

6. The blood which remains in the alveoli, and with the pneumonia infiltration undergoes cheesy metamorphosis, not unfrequently occasions an eruption of miliary tubercles.—*Med. and Surg. Reporter.*

